# Thermal Ecology and Forensic Implications of Blow Fly (Family: Calliphoridae) Maggot Mass Dynamics: A Review

**DOI:** 10.3390/insects16101018

**Published:** 2025-10-01

**Authors:** Akomavo Fabrice Gbenonsi, Leon Higley

**Affiliations:** School of Natural Resources, University of Nebraska-Lincoln, Lincoln, NE 68588, USA; gbenonsifab@yahoo.com

**Keywords:** maggot mass, postmortem interval, thermoregulation, heat shock proteins, volatile organic compounds, decomposition

## Abstract

**Simple Summary:**

When animals die in nature, blow flies are often the first insects to arrive. Their larvae (maggots) gather in large groups, forming maggot masses that produce heat and accelerate decomposition. This process helps recycle nutrients, but it also creates challenges for scientists who use these insects to estimate the time since a body has been dead in criminal investigations. This review explains how maggot masses work, why the larvae cluster together, how they generate heat, and how they compete or cooperate. We found that these masses create unique hotspots that help the larvae grow faster but can also make it harder to predict their age accurately. Understanding these dynamics is important for both learning how nature breaks down dead matter and improving crime scene investigations. This review highlights the need for improved tools to account for maggot mass effects when estimating the time of death. This knowledge could help solve crimes more accurately while also teaching us about the role insects play in maintaining clean ecosystems. Future research should investigate how climate change may impact these processes, as warmer temperatures could alter blow fly behavior and decomposition rates.

**Abstract:**

Blow flies (Diptera: Calliphoridae) play a crucial role in the decomposition process and serve as important forensic indicators due to their predictable colonization patterns. This review focuses on the dynamics of maggot masses, highlighting their ecological roles, thermoregulation, and implications for forensics. We summarize data on the self-organizing behavior of maggot masses, which is influenced by chemical cues and environmental factors. These masses can generate internal temperatures that exceed ambient levels by 10–20 °C, accelerating larval growth and impacting competition among individuals. This localized heating complicates the estimation of the postmortem interval (PMI), as traditional models may not take these thermal influences into account. Furthermore, maggot masses contribute significantly to nutrient cycling and soil enrichment, while the behavior of the larvae includes both cooperation and competition, which is influenced by the species composition present. This review highlights challenges in PMI estimation due to heat production but also discusses advancements in molecular tools and thermal modeling that enhance accuracy. Ultimately, we identify knowledge gaps regarding species diversity, microbial interactions, and environmental variability that impact mass dynamics, suggesting future research avenues that could enhance ecological understanding and forensic applications.

## 1. Introduction

As significant contributors to the decomposition of vertebrate remains, blow flies (Diptera: Calliphoridae) are essential to ecosystem functioning and nutrient recycling [1,2,3]. Because of the Volatile Organic Chemicals (VOCs) released during the early phases of decomposition, blow flies are frequently the first to arrive on a body [4,5,6]. In legal investigations, species like *Phormia regina*, *Calliphora vicina*, and *Lucilia sericata* are significant because of their vast host range, consistent developmental rates, and capacity to flourish in diverse settings [7,8]. These species’ larvae feed on decaying tissue and aggregate into dense clusters called maggot masses, which substantially impact the rate and pattern of decomposition [9]. Maggots are the soft-bodied larvae of blow flies in the Cyclorrhapha, often used to describe larvae of decomposers like the Calliphoridae, Muscidae, and Sarcophagidae.

Maggot masses are dynamic, self-organizing formations that display complex behaviors, including coordinated feeding, collective locomotion, and thermoregulation [10,11]. These groups, which may range in size from hundreds to thousands of larvae, provide a microenvironment that is very different from the ecosystem around them [12]. The heat generated by maggot masses often exceeds ambient temperatures by 10–20 °C. This results from metabolic activity and can accelerate larval development and decomposition rates [9,13,14]. However, the high density of larvae in these masses causes intense competition for resources like food and oxygen, influencing survival rates and developmental outcomes [10,15]. Forensic entomology, especially in determining the postmortem interval (PMI), is significantly impacted by the activity and survival of blow fly larvae within maggot masses [1,16]. Due to the special conditions in masses, general PMI computation may have to be revised as it relies on the anticipated rates of blow fly growth [9,17]. Therefore, it is crucial to understand the elements that affect maggot mass dynamics to increase the precision of forensic investigations.

Although understanding maggot masses is essential for both ecology and forensics, little is known about how aggregations affect the survival of the principal forensic blow fly species. In the following, we summarize the literature on the dynamics of maggot masses and how they affect blow fly survival and development. This review will look at the relationships between variables, including temperature, density, and species interactions.

## 2. Mechanisms of Insect Aggregation

Insect aggregations have significant ecological and evolutionary benefits that result from social interactions, chemical communication, and environmental signals.

It is well known that pheromones play a crucial role in coordinating group activity among insects [18]. Aggregation of insects is widespread in many different taxa, including solitary species that display momentarily collective behavior to serve specific purposes. Studies have shown that under the influence of competition, tree-killing bark beetles *Dendroctonus* spp. produce aggregation pheromones to target healthier plants. This behavior encourages conspecifics and guarantees that the group’s combined strength surpasses the tree’s defenses [19]. Likewise, gregarious locusts, *Schistocerca gregaria*, have a well-documented phase of polyphenism where they shift from solitary to gregarious phases in response to population density and environmental factors, a process influenced by changes in serotonin levels in the brain [20]. Apart from pheromones, VOCs released by plants or decaying matter can also trigger aggregation. Studies have revealed that fruit flies, *Drosophila melanogaster,* are attracted to acetic acid from fermenting fruits, as well as 2-phenylethanol and acetoin, serving as synergistic compounds [21]. Among the necrophagous insect community, VOCs represent a major signal of carrion presence, especially among blowflies. Numerous studies have been focused on identifying the VOCs responsible to blowfies’ attraction to carrion. For instance, dimethyl disulfide and butan-1-ol have been uncovered as attractive to *Lucilia sericata* [22].

Environmental aspects like temperature, light, and resource availability are crucial to insect aggregation. While volatile organic compounds (VOCs) drive initial insect-host interactions, temperature critically regulates their emission dynamics and insect responsiveness. This temperature dependence extends beyond chemical ecology, some insects, like honeybees *Apis mellifera*, actively manipulate thermal conditions to optimize survival. Individual bees generate heat using muscle activity, and the group’s collective behavior helps maintain an optimal temperature for brood rearing [23]. Similarly, tent caterpillars (*Malacosoma* spp.) create silk tents that provide thermal advantages, enabling larvae to maintain higher body temperatures and speed up development [24]. Beyond temperature, light serves as a critical environmental signal for insect aggregation. In fireflies (Lampyridae), mating success depends on coordinated flashing displays. The precision of this behavior is modulated by temperature and ambient light conditions [25].

The availability of resources could sometimes drive insect aggregation. For instance, their feeding efficiency and reproductive success are improved when ladybird beetles (*Coccinella septempunctata*) congregate in locations with a high density of prey [26]. Similarly, dung beetles (*Scarabaeus* spp.) congregate near dung pats to compete for mates and resources [27]. Paradoxically, these gatherings promote cooperation through communal burial, which minimizes interspecific pressure and nutrient loss.

Social interactions are another key mechanism driving insect aggregation. Many species show flexible leadership dynamics during collective movement. In social caterpillars *Eriogaster lanestris*, nutritionally advantaged individuals initiate group relocation to new feeding sites [28], while sawfly larvae *Perga affinis* show rotating leadership during foraging, with multiple individuals alternately guiding group movement [29]. These roles are context-dependent, often modulated by individual nutritional state and resource availability.

Such aggregations confer significant survival benefits through collective predator avoidance strategies. Monarch butterflies, *Danaus plexippus*, migratory clusters create sensory confusion for predators, statistically reducing individual predation risk through the dilution effect [30]. Similarly, fishfly swarms, *Chauliodes* spp., employ collective motion patterns that disrupt predator targeting efficiency [31].

Aggregations can also enhance resource utilization by allowing individuals to exploit resources more efficiently. Termites form large colonies that work collectively to break down cellulose, a resource that would be difficult for individual termites to exploit independently [32]. Likewise, blow flies produce heat-producing maggot masses that speed up the decomposition of decaying tissue and enable the larvae to utilize the resource more effectively [33]. Unlike the largely cooperative aggregations observed in prey-rich or migratory systems, aggregation in carrion insect communities is strongly shaped by competition. Blow flies and *Necrodes* beetles (Silphidae) exemplify this dynamic through contrasting yet interactive strategies. Blow flies rapidly colonize fresh carcasses in response to VOCs, concentrating oviposition and producing dense larval aggregations that accelerate tissue degradation and enhance monopolization of resources. In contrast, *Necrodes* beetles typically aggregate later, preferentially on carcasses where fly performance is reduced, guided by carrion-derived cues and conspecific signals. Their aggregations are reinforced by interference, as adults actively kill feeding fly larvae. Thereby they diminish fly dominance. Thus, in carrion systems, aggregation functions not only as a response to ephemeral resources but also as a mechanism mediating the balance between exploitative and interference competition [34].

## 3. Formation and Composition of Maggot Masses

A combination of environmental and biological factors influences maggot mass formation. Temperature and humidity are critical, as they directly affect the activity of adult blow flies and the development of larvae [9,35,36,37]. Blow flies are attracted to decomposing organic matter by VOCs emitted during the early stages of decomposition [2,38]. Once eggs are laid in natural orifices, wounds, or other moist areas, they hatch into larvae that aggregate into masses to optimize feeding and thermoregulation [10]. Another factor is the nature of substrate; carrion and wounds offer the best circumstances for the growth of larvae [7]. Furthermore, the size and makeup of maggot masses can be influenced by rival necrophagous insects [39].

The distinct actions of several blow fly species aid in creating maggot masses. While *C. vicina* and *P. regina* are more resistant to lower temperatures, *L. sericata* is known for its quick colonization of cadavers and affinity for damp settings [40]. Variations in maggots’ mass composition and dynamics can result from these species-specific variances in egg-laying behavior, larval growth, and aggregation tendencies [7]. Understanding these characteristics is required to predict maggot mass formation and determine how it affects decomposition rates.

Blow fly larvae aggregate into masses for several reasons, including thermoregulation, feeding efficiency, and predator protection [33]. Although large larval densities might result in higher competition for resources, these collective actions are thought to improve the survival and development of individual larvae [10]. However, [41] demonstrated that at excessively high larval densities, the negative effects of competition can outweigh the benefits of aggregation. Their study shows that beyond a certain threshold, increased density leads to elevated mortality, reduced pupal weight, and lower adult emergence rates.

Substrate type and environmental factors significantly influence maggot mass development and composition. For instance, temperature, humidity, and carrion availability differences may change maggot masses in urban and rural settings [2]. Indoor and outdoor conditions can also impact maggot mass dynamics and blow fly colonization patterns [39].

In conclusion, a complex interaction of biological, behavioral, and environmental variables shapes the genesis and makeup of maggot masses. However, despite their relevance, these dynamics remain underrepresented in current forensic protocols, contributing to potential inaccuracies in PMI estimations. Future studies should concentrate on how species-specific behaviors and chemical cues promote larval aggregation and how substrate and environmental changes affect the development of maggot masses.

## 4. Thermoregulation in a Maggot Mass

Maggot masses are renowned for producing large amounts of heat; this phenomenon is sometimes called “self-heating” or “endothermy” [16]. The metabolic activity of thousands of feeding larvae produces this heat as a byproduct. These larvae break down organic material collectively and release energy in the form of heat [10]. The temperature within a maggot mass can exceed ambient temperatures by 10–20 °C, with some masses reaching internal temperatures of 40 °C or higher [13,14,16]. Blow flies are thought to have a competitive advantage over other decomposers because of the unique microenvironment that this high temperature produces, which speeds up larval growth and decomposition rates [12].

Both biological and behavioral factors mediate maggot masses’ capacity to control their temperature. Larval metabolism produces heat that may increase mass temperatures, speeding up growth and giving an edge over competitors [9]. The leading cause of temperature rise is metabolic heat generation, which occurs when larvae break down proteins and lipids in decaying tissue [10]. Dense aggregations of *L. sericata* larvae generate metabolic heat, elevating mass temperatures up to 20 °C above ambient, with maximal heating (ΔT 8–14 °C) occurring at 22–25 °C ambient. This effect requires ≥50 g (~1450 larvae) to initiate and is self-regulated to avoid lethal overheating (>40 °C) through behavioral thermoregulation [9]. *Chrysomya megacephala* larvae demonstrate behavioral thermoregulation with coordinated positional rotations within the mass to distribute thermal exposure. Individuals cycle between warmer core regions and cooler peripheries in response to thermal gradients [12]. At the molecular level, small heat shock proteins (e.g., SmHsp22.2 and SmHsp26.7) seem important for insect thermoregulation during diapause, with species-specific expression patterns. In *Stiodiplosis mosellana*, these sHsps are upregulated under moderate thermal stress (35–40 °C or −10 °C) but fail to counteract extreme temperatures, and their knockdown significantly impairs cold tolerance [42]. This multi-level thermoregulatory system optimizes developmental conditions while mitigating thermal stress. Larval clustering is believed to be a form of behavioral adaptation essential for heat distribution and retention. To minimize localized overheating, larvae frequently move in synchronized waves that disperse heat throughout the bulk [16,33]. Furthermore, when bacteria and fungi decompose organic waste and release energy, microbial activity inside the mass adds to heat generation [43]. These methods allow maggot masses to sustain steady and ideal temperature conditions for larval development. Because heat speeds up metabolic rates, blow fly larvae may grow and develop more quickly [16]. A maggot mass’s core, where temperatures are most significant, may see faster larval development than its perimeter [41]. Because it can make estimating the PMI more complex, a mass’s rapid growth substantially impacts forensic entomology. Forensic entomologists must account for the thermal dynamics of maggot masses when analyzing insect evidence, as failure to do so can lead to inaccurate PMI estimates [1,44]. Traditional forensic models often rely on ambient temperatures and overlook the significant thermal contribution of maggot masses, potentially introducing error into PMI estimations.

From an ecological perspective, the high temperatures in maggot masses speed up decomposition, promoting ecosystem energy and nutrient cycling [2]. However, the heat might also prevent the growth of competing species that could otherwise use the same resources, such as fungi and bacteria [10]. The thermoregulatory abilities of maggot masses not only influence larval development and forensic applications but also play a key role in their ecological function.

## 5. Ecological Role of Maggot Masses

Maggot masses are necessary for the decomposition of organic waste, particularly carrion. The enzymatic activity of blow fly larvae facilitates the release of nutrients into the environment and accelerates tissue breakdown [45]. According to [46], these enzymes, including lipases and proteases, convert lipids and proteins into simpler substances that the larvae consume or discharge into the soil. By swiftly clearing decaying organic materials from the environment, maggot masses’ fast decomposition recycles nutrients and lowers the danger of disease transmission [47].

In terrestrial ecosystems, maggot masses play a crucial role in the cycling of nutrients. Through their excretions and the breakdown of their bodies, larvae that consume carrion release nitrogen, phosphorus, and other vital nutrients into the soil [48]. Initially, the high nitrogen increase associated with decompositional fluids is toxic to plants. Later, a positive feedback loop that increases ecosystem production is created by this nutrient enrichment, which also stimulates plant development and increases soil fertility [49]. Increased soil nutrient levels have been seen in regions with high maggot mass activity, which may impact the variety and composition of plant communities [50,51].

Various insect species interact with maggot masses, such as rival scavengers, predators, and parasitoids. These interactions can influence ecological communities’ dynamics and structure. Some scavengers, like beetles, may be repelled by the heat produced by maggot masses, while others, like ants, may be drawn to them [52]. Predators like birds and small animals may eat blow fly larvae, while parasitoids like wasps target the larvae to reproduce [16,53]. These intricate relationships demonstrate maggot masses’ importance to the food webs linked with carrion.

Maggot masses have an ecological impact that goes beyond nitrogen cycling and decomposition to affect the dynamics of larger ecosystems. Supplementing the soil with nutrients enhances plant development and contributes to ecosystems’ general stability and health [54]. The quick clearance of carrion by maggot masses also limits the resources available to competing decomposers like fungi and bacteria, which can change the composition and function of microbial communities [39].

## 6. Forensic Application of Maggot Masses

To precisely determine the PMI, forensic entomologists can reconstruct the chronology of insect colonization by examining the size, age, and species composition of maggot masses [16,55]. This technique is fundamental to forensic investigations because it is based on the idea that, at particular temperatures, blow fly growth follows predictable patterns. Maggot masses provide several difficulties when estimating the PMI. Variations in the temperature of the maggot mass and its surroundings can substantially affect the pace of growth and may result in errors [10,13,56].

Additionally, it is more difficult to identify species and interpret data when several blow fly species are present, each with a different pace of growth [57,58]. Moreover, several species of blow flies, each with a unique development rate, make it more difficult to identify species and interpret data [57,58]. The thermoregulatory behavior of maggot masses further complicates PMI computations, as the heat produced by the larvae can create microclimates different from the surrounding environment. These difficulties demonstrate the need for careful data gathering and analysis in the forensic entomology area.

Maggots have been used as vital evidence in helping to recreate crime scenes and estimate the PMI. However, precise species identification and developmental data are essential [59]. Recent developments in forensic entomology have increased the precision and dependability of maggot mass analysis. Molecular methods, including DNA barcoding, have made it possible to identify species precisely, even when physical traits are unclear [60,61]. PMI assessment has been further enhanced by improved models that consider environmental variability and maggot mass thermoregulation [1].

When modeling maggot mass temperatures, based on our findings [41], it is a mistake to use the actual maggot mass temperature. We make this observation because experimental evidence in development in maggot masses shows that competitive effects can actually delay development relative to predictions from maggot mass temperature [41]. At this point, the most conservative recommendation is to use ambient temperatures for calculating maggot development for growth outside and inside maggot masses. A failure to use such an approach will lead to overestimates in the speed of maggot development.

## 7. Competition vs. Cooperation in Maggot Masses

In nature, species interactions often exist in a delicate balance where the same ecological factors that create opportunities for coexistence can also drive competitive exclusion. Carrion-feeding insect species community vary across different environments [62]. This variation may be influenced by factors such as habitat [63], food availability, the presence or absence of other insect species [64], and seasonal changes [17,65]. Researchers have found that the rate of carcass loss can also vary depending on the size of the corpse and model (human versus pig, for example) [66]. Maggot masses are dynamic systems where competition and cooperation likely coexist, and they often blur the line between these two concepts. Competition is a potentially dominant force in maggot masses, particularly in high-density populations. Both intra-species and inter-species competition occur as larvae compete for relatively limited resources, such as food and space [10]. Competition shortens larval development, which has implications for postmortem interval estimations [67]. Sharing the same ecosystem with other blow flies, *Chrysomya rufifacies* demonstrates an ultimate competitive advantage by preying on others when food is scarce [68]. The succession of colonizing species has also been reported to avoid competition among species [69]. Community assembly is driven by species colonization rather than competitive exclusion, suggesting that succession mechanisms promote coexistence by lowering direct competition [70]. The relationship between *Ch. megacephala* and *Chrysomya pinguis* illustrates this duality, as closely related species partition carrion resources through divergent thermal adaptations. Zang et al. [71] demonstrate that both exploit carcasses, a transient and patchily distributed resource, yet temporal and spatial segregation minimizes direct conflict. *Ch. megacephala* dominates under warmer conditions, prevailing during summer months and at lower elevations. In contrast, *Ch. pinguis* prospers in cooler environments, displacing its congener in winter or at higher altitudes. Temperature gradients thus act as natural boundaries, maintaining equilibrium without overt confrontation. At intermediate temperatures, however, this balance breaks down. At 30 °C, *Ch. megacephala* gains a competitive advantage, suppressing *Ch. pinguis* eclosion. These dynamics demonstrate how environmental thresholds mediate the shift from passive resource partitioning to active competition. The absence of direct interference, combined with niche differentiation, demonstrate through this study that coexistence may be sustained through physiological trade-offs rather than explicit cooperation or conflict. To properly comprehend current competition or the factors contributing to this occurrence, one must know how much food larvae require for optimum development. Although direct measurements of the food quantity required by a single maggot for optimal development are lacking, studies indicate that larval density, even under adequate feeding conditions can slow development due to space and thermal constraints [41]. However, food insufficiency in crowded environments leads to premature development, reduced adult size, and lower fitness [72,73]. Although competition is standard, maggot masses often display cooperative behaviors that improve survival as a group. Chemical cues, including aggregation pheromones, influence these behaviors by drawing larvae together in dense aggregations [74]. The whole mass benefits from the favorable microenvironment created by the heat produced by larval metabolism, which speeds up growth and discourages predators [10]. A study showed that *Calliphora vomitoria* and *L. sericata* larvae prefer areas marked by previous larval presence, whether from their species or another. This marking effect is more potent when more larvae are present. *L. sericata* prefers conspecific cues over heterospecific ones, but only at high concentrations. This suggests that larval markings help them aggregate, potentially leading to interspecific groupings [74]. Similarly, intra- or inter-larval cooperation plays a key role in key choices. *L. sericata* and *C. vicina* larvae show lower growth and survival on rotten food, but mixing species reduces this adverse effect, leading to larger adult mass. Larvae preferred fresh liver in meal choice tests, and accuracy improved with increasing collective size. However, the preference for rotting food changed when another species was present [75]. This implies that interactions between species affect how resources are used, which may make it possible to access new food sources by creating niches.

The distinction between cooperation and competition can be unclear in maggot masses since both behaviors might co-occur. The balance between competition and cooperation is further demonstrated by the possibility that the dominant species in mixed-species assemblages may profit from the heat produced by subordinate species. These interactions demonstrate how adaptable blow fly larvae are, exhibiting cooperative and competitive behaviors in response to changing environmental factors and resource availability. Cooperative behaviors, such as coordinated feeding and thermoregulation, predominate at low densities because the larvae gain from the group benefits of aggregation. However, competition for resources becomes fiercer as density rises, which raises mortality and causes a change in behavior toward competition [10]. Environmental elements that affect resource availability and larval growth, such as temperature and humidity, also impact competition [2]. Since these dynamics affect larval growth, decomposition rates, and PMI estimates, understanding them is essential for ecological research and forensic applications. To learn more about when and why the boundary between cooperation and competition changes, future studies should concentrate on the physiological and chemical processes that underlie these behaviors, especially in mixed-species masses.

## 8. Adaptive Plasticity and Heat Shock Protein Expression in Blowflies Facing Thermal Stress

Heat shock proteins (HSPs) are conserved and found in all organisms, playing an important role in maintaining cellular proteostasis and protecting against stress. These proteins are classified by their molecular weight and function as molecular chaperones. They assist in the folding, refolding, and degradation of other proteins, as well as in regulating cell signaling, the cell cycle, and apoptosis [76].

In blowflies, HSPs are essential for thermoregulation, enabling these insects to survive and function in extreme and fluctuating temperature conditions typical of their ecological environments. The activation of heat shock proteins in response to thermal stress serves as a key adaptive mechanism, supporting protein stability and preventing cellular damage [77].

Among the HSP families, HSP70 is prominently expressed in response to elevated temperatures in insects, including blowflies. It plays a central role in the refolding of denatured proteins and in preventing protein aggregation under heat stress. Studies have shown that the expression of HSP70 in blowflies increases significantly in response to high temperatures, supporting larval survival and development in thermally challenging environments [78]. Similarly, small heat shock proteins (sHSPs), which function as early responders to heat shock, play a crucial role in stabilizing partially denatured proteins and maintaining cytoskeletal integrity during heat exposure [79].

The thermoregulation capabilities of blowflies are closely linked to the spatial and temporal regulation of heat shock protein (HSP) gene expression. Research shows that blowfly larvae adjust HSP expression based on the intensity and duration of thermal exposure. This indicates a finely tuned regulatory system that enhances their thermal resilience [80]. This adaptive plasticity enables blowflies to thrive in a variety of thermally dynamic microhabitats, such as decomposing carcasses, where temperatures can change drastically over short distances and periods.

Research on related dipterans, such as the sarcophagid *Sarcophaga crassipalpis*, indicates that an increase in HSP70 levels enhances cold hardiness during diapause. This finding suggests that variations in HSP expression could be the basis for different thermoregulatory strategies among species [80]. These variations are particularly crucial in light of climate change, as blowflies must adapt to shifting thermal conditions that impact their life history traits.

## 9. Conclusions

Maggot masses involve a complex interaction of biological, behavioral, and environmental factors that significantly affect the survival, development, and ecological functions of blow flies. Aggregated larval ability to control temperature, influenced by metabolic heat and collaborative behaviors, aids in larval development; however, it also complicates forensic assessments of PMI due to differences in microclimates. Resource competition among the groups further affects the outcomes for larvae. In addition, heat shock proteins are vital for coping with thermal stress. Despite their ecological and forensic importance, significant knowledge gaps remain, especially concerning the specific signals that various species utilize for aggregation and the effects of environmental changes on these interactions. Future research should focus on these aspects to enhance forensic models and improve our understanding of blow fly ecology in the context of climate change.

## Data Availability

No new data were created or analyzed in this study. Data sharing is not applicable to this article.
